# Alcohol Consumption Patterns and Traditional Beverages Associated with Hypertension Subtypes

**DOI:** 10.3390/medsci14010060

**Published:** 2026-01-28

**Authors:** Fiorella E. Zuzunaga-Montoya, Jhosmer Ballena-Caicedo, Oriana Rivera-Lozada, Mario J. Valladares-Garrido, Jean Pierre Eduardo Zila Velasque, Víctor Juan Vera-Ponce

**Affiliations:** 1 Escuela de Medicina Humana, Universidad Continental, Lima 00051, Peru; 2 Facultad de Medicina (FAMED), Universidad Nacional Toribio Rodríguez de Mendoza de Amazonas (UNTRM), Chachapoyas 01000, Peru; 3 Escuela de Medicina Humana, Universidad Señor de Sipán, Chiclayo 14000, Peru; 4 Red Latinoamericana de Medicina en la Altitud e Investigación (REDLAMAI), Cerro de Pasco 19000, Peru; 5 EpiHealth Research Center for Epidemiology and Public Health, Universidad Peruana Cayetano Heredia, UPCH, Lima 15001, Peru

**Keywords:** hypertension, alcohol consumption, public health, Peru

## Abstract

**Introduction**: Alcohol consumption is a significant risk factor for hypertension (HTN), a prevalent condition that substantially affects cardiovascular health. In Peru, where various traditional alcoholic beverages exist, the relationship between alcohol consumption and HTN has not been fully explored. **Objective**: To determine the association between different patterns of alcohol consumption, types of beverages, and various types of HTN. **Methods**: This cross-sectional analytical study utilized data from the Peruvian Demographic and Family Health Survey (2018–2023), including 236,243 adults (55.95% male; mean age: 41.06 years). General HTN, isolated systolic hypertension (ISH), isolated diastolic hypertension (IDH), and systolic-diastolic hypertension (SDH) were evaluated. Alcohol consumption was assessed through self-reported questionnaires evaluating consumption pattern (non-excessive vs. excessive), intensity (light, moderate, heavy), consistency (intermittent vs. consistent), and primary beverage type, including both commercial and traditional Peruvian drinks. **Results**: Excessive alcohol consumption was significantly associated with an increased risk of HTN (aPR: 1.19, 95% CI: 1.07–1.31), IDH (aPR: 1.61, 95% CI: 1.20–2.16), and SDH (aPR: 1.45, 95% CI: 1.18–1.78). Excessive alcohol consumption was significantly associated with an increased risk of HTN (aPR: 1.19, 95% CI: 1.07–1.31), IDH (aPR: 1.61, 95% CI: 1.20–2.16), and SDH (aPR: 1.45, 95% CI: 1.18–1.78). Consumption of traditional beverages such as chicha and Masato was associated with an elevated risk of various types of HTN. In contrast, wine consumption demonstrated a protective association against general HTN and IDH. **Conclusions**: Alcohol consumption patterns and beverage types have differential effects on HTN risk in the Peruvian population. These findings underscore the need for culturally adapted prevention strategies and more nuanced public health recommendations regarding alcohol consumption in Peru.

## 1. Introduction

Hypertension (HTN) is a significant risk factor for cardiovascular diseases, which are the leading cause of death worldwide [1]. It is estimated that HTN affects more than 1.3 billion people globally, with a prevalence that increases with age [2]. Recent studies have reported a prevalence of HTN of approximately 23% in the adult population in Peru, with significant variations across geographical regions [3].

Alcohol consumption is a well-established modifiable risk factor for HTN [4]. However, the relationship between alcohol consumption and HTN is complex and may vary depending on the pattern of consumption and the type of alcoholic beverage [5]. While excessive alcohol consumption is consistently associated with an increased risk of HTN, some studies have suggested that moderate consumption of certain alcoholic drinks, such as red wine, might have different effects on cardiovascular health [6].

The majority of studies on alcohol and HTN have focused on Western populations and alcoholic beverages common in these regions [7]. However, in countries like Peru, there exists a diversity of traditional alcoholic drinks, such as chicha, masato, and yonque, whose impact on cardiovascular health has been poorly studied [3]. Moreover, alcohol consumption patterns can vary significantly across cultures and geographical regions, underscoring the importance of context-specific studies [7].

Furthermore, HTN is not a homogeneous condition. Different subtypes can be distinguished, including isolated systolic hypertension (ISH), isolated diastolic hypertension (IDH), and systolic-diastolic hypertension (SDH), each with its own clinical and prognostic implications [8,9]. Recent studies have suggested that these HTN subtypes might have different risk factors and underlying pathophysiological mechanisms [10,11,12,13]. However, the specific relationship between various types of alcoholic beverages and these HTN subtypes has not been exhaustively explored.

In this context, the present study aims to examine the relationship between different alcohol consumption patterns, including traditional Peruvian beverages, and the distinct subtypes of HTN in a representative sample of the Peruvian population. This approach will allow for a more nuanced understanding of the relationship between alcohol consumption and HTN, which could have important implications for HTN prevention and management strategies in the Peruvian context and, potentially, in other Andean countries.

## 2. Methodology

### 2.1. Study Design and Context

This study employs a cross-sectional, analytical approach, utilizing secondary data from Peru’s Demographic and Family Health Survey (DHS). Our analysis focuses on survey data collected from 2018 to 2023.

The DHS, conducted annually by Peru’s National Institute of Statistics and Informatics (INEI in Spanish), provides a comprehensive source of information on various health and demographic indicators.

### 2.2. Population, Sample, and Eligibility Criteria

The target population for this study comprises all adults aged 18 years or older residing in private households in Peru during 2023. DHS employs a probabilistic, stratified, multi-stage sampling design, ensuring adequate national representation by area of residence (urban and rural) and by natural regions (Coast, Highlands, and Jungle).

For our specific analysis, we included all adult participants with complete data on alcohol consumption and systolic (SBP) and diastolic blood pressure (DBP) measurements. To ensure data validity and minimize potential biases, we applied the following exclusion criteria: (1) Pregnant women were excluded from the analysis due to pregnancy-associated physiological changes in blood pressure. (2) To ensure the reliability of blood pressure data, cases with extreme or implausible values were eliminated, following World Health Organization (WHO) recommendations for surveillance of non-communicable disease risk factors. Records with SBP equal to or greater than 250 mmHg and cases with DBP below 50 mmHg were excluded. (3) For the analysis of alcohol consumption, cases with inconsistent responses or implausible values in questions related to alcoholic beverage consumption were excluded.

These exclusion criteria are based on biologically plausible ranges. They are consistent with standard practices in large-scale epidemiological studies, thus ensuring the quality and reliability of our analyses on the relationship between alcohol consumption and different types of HTN in the Peruvian population.

### 2.3. Variables and Measurement

The main variables were defined as follows [8,11,14]:

1. General HTN was defined as SBP ≥ 140 mmHg and/or DBP ≥ 90 mmHg.
2. ISH was defined as SBP ≥ 140 mmHg but with DBP < 90 mmHg, regardless of previous HTN diagnosis.
3. IDH was defined as DBP ≥ 90 mmHg but with SBP < 140 mmHg.
4. SDH was defined as SBP ≥ 140 mmHg and DBP ≥ 90 mmHg.

The primary independent variable was alcohol consumption, which was evaluated in four distinct ways to capture different aspects:

1. Consumption pattern was classified into three categories based on self-reported intake patterns. The first category included individuals who had never consumed alcohol or had not done so in the past year. The second category comprised those who had consumed alcohol in the last 30 days without engaging in excessive consumption. The third category, defined as excessive consumption, encompassed participants who reported at least one episode of consuming five or more drinks for men or four or more for women on a single occasion during the past 30 days [15].
2. Consumption intensity was determined based on the number of standard drinks consumed on a typical drinking day during the past 30 days. Three levels were established: light consumption (1–2 standard drinks per occasion), moderate (3–4 standard drinks per occasion), and high (5 or more standard drinks per occasion). For this purpose, a standard drink was defined as containing approximately 14 g of pure alcohol, following international guidelines for standardizing alcohol consumption measures.
3. Primary beverage type was determined by asking participants which alcoholic beverage they consumed most frequently in the past 12 months. Options included common beverages such as beer and wine and traditional Peruvian drinks like chicha, masato, and yonque. Categories for whiskey and other beverages were also included. In cases where participants reported consuming multiple beverages with equal frequency, they were classified in the “No clear preference” category, thus acknowledging the diversity in consumption patterns.
4. Consumption consistency was evaluated based on the frequency of consumption reported by participants during the past 12 months. Two categories were established: inconsistent consumption, which included those who reported consuming alcohol less than once a month or only on special occasions, and consistent consumption, encompassing participants who reported consuming alcohol at least once a month regularly throughout the year. This classification distinguishes between occasional consumption patterns and those that are more habitual or entrenched.

For description and subsequent analyses, several covariates were included in the Study and selected for their potential influence on the relationship between both variables. Age was measured in completed years, recognizing its significant impact on the risk of HTN. Sex was categorized as male or female, given the known differences in alcohol consumption patterns and HTN prevalence between these groups. Residence was classified as urban or rural, considering lifestyle variations, alcohol consumption patterns, and access to health services between these environments. The wealth index was divided into five categories: very poor, poor, middle, rich, and very rich, allowing for evaluation of how socioeconomic status may moderate the relationship between both variables. The natural region of residence (Metropolitan Lima, Rest of Coast, Highlands, Jungle) was included to capture geographical and cultural differences. The altitude of the residence was measured in meters above sea level. Finally, smoking status was categorized into four groups: never smoked, former smoker, occasional smoker, and daily smoker, reflecting the importance of smoking as a cardiovascular risk factor and its frequent coexistence with alcohol consumption.

### 2.4. Procedures

The DHS in Peru has modernized its data collection methods, using digital tablets since 2016. This collection is carried out following internationally standardized and validated protocols. Trained INEI personnel conduct interviews and measurements in selected households, ensuring the quality and consistency of the obtained data.

For blood pressure measurement, a crucial element in our study on different types of HTN is that automatic digital sphygmomanometers are used, which are regularly calibrated to ensure accuracy. The procedure involves taking three blood pressure measurements with the participant seated after a rest period of at least 5 min. To minimize the “white coat” effect, the average of the last two measurements is used in the analysis, discarding the first one.

Information on alcohol consumption, our primary independent variable, was collected through a structured questionnaire during the interview. To assess recent consumption patterns, participants were asked about their alcohol consumption in the last 12 months and the previous 30 days, including specific questions about frequency and quantity of consumption. Consumption intensity was evaluated by inquiring about the typical number of drinks consumed on a drinking day. For the primary beverage type, participants were asked to indicate which alcoholic beverage they consumed most frequently, offering options that included traditional Peruvian drinks. Consumption consistency was determined from questions about the frequency of consumption throughout the year.

To ensure the accuracy of responses related to alcohol consumption, interviewers were trained to use visual aids illustrating standard drink sizes and to provide clear examples of what constitutes a “standard drink” in the Peruvian context. Additionally, verification questions were implemented to detect inconsistencies in responses about alcohol consumption.

Anthropometric measurements and information on sociodemographic, economic, and lifestyle variables were collected following standardized protocols. All participants provided informed consent before participating in the survey. Furthermore, the collected data underwent a rigorous quality control process, which included direct supervision of a sample of the interviews and repeated measurements in a subsample of participants to ensure the reliability of the data used in our analysis.

### 2.5. Statistical Analysis

For our study, a comprehensive statistical analysis was conducted in several stages. Initially, a descriptive analysis of all study variables was performed. For categorical variables, frequencies and percentages were calculated. Means and standard deviations were obtained for numerical variables. Subsequently, a bivariate descriptive analysis was carried out to examine the distribution of variables about different types of HTN.

A Poisson regression model with robust variance was chosen to evaluate the association between variables of interest. This approach was selected due to its ability to directly estimate adjusted prevalence ratios (aPR), more interpretable in cross-sectional studies with frequent outcomes such as HTN. Separate models were constructed for each type of HTN and each measure of alcohol consumption.

To guide the selection of covariates for adjustment and minimize confounding bias, a Directed Acyclic Graph (DAG) was developed based on existing scientific evidence for each outcome variable. This DAG allowed us to identify the minimal sufficient adjustment set, which included age, sex, residence (urban/rural), wealth index, natural region, altitude, and smoking status. This ensures that the included variables were genuine confounders and not mediators or colliders in the causal pathway between alcohol consumption and HTN.

Each model was adjusted for all previously mentioned covariates, with particular attention to age and altitude, which were modeled using restricted cubic splines (RCS) to capture potential non-linear relationships with HTN outcomes. Other covariates included were sex, residence, wealth index, natural region, and smoking status, considering that each could act as an essential confounding variable in the relationship between the main variables.

All statistical analyses were performed using Stata software version 17.0 (StataCorp, College Station, TX, USA). For the aforementioned variables, restricted cubic splines were implemented using Stata’s ‘mkspline’ command. The gtsummary package was used to present descriptive statistics and regression model results, which facilitates the efficient and standardized creation of statistical summary and results tables. Graphs, including visual representations of non-linear effects of age and altitude, were generated using the ggplot2 package in R version 4.0.3.

It is important to emphasize that all analyses were conducted considering the complex sampling design, using the sampling weights provided by DHS to ensure the representativeness of results at the national level. Results were considered statistically significant when the 95% confidence interval (CI) of the aPR did not cross unity.

### 2.6. Ethical Considerations

This study is based on a secondary analysis of data provided by DHS, a publicly accessible information source. INEI makes these data available after a rigorous anonymization process, removing personal identifiers to ensure participant confidentiality. Since our research did not involve direct contact with subjects and worked exclusively with anonymous data, additional evaluation by an ethics committee was not required to proceed with this secondary analysis. Nevertheless, it is crucial to emphasize that our research team has conducted this study in strict adherence to the ethical principles established in the Declaration of Helsinki.

## 3. Results

The study sample included 236,243 participants, with a slightly higher proportion of males (55.95%) than females (44.05%). The mean age of participants was 41.06 years (SD 16.34). The majority resided in urban areas (78.91%) and had secondary (43.88%) or higher (34.67%) education. The distribution of the wealth index was relatively balanced across quintiles. The overall prevalence of HTN was 13.15%, with variations according to type: 7.47% for ISH, 1.73% for IDH, and 3.58% for SDH. The majority of participants (78.44%) had never smoked (Table 1).

**Figure 1:**
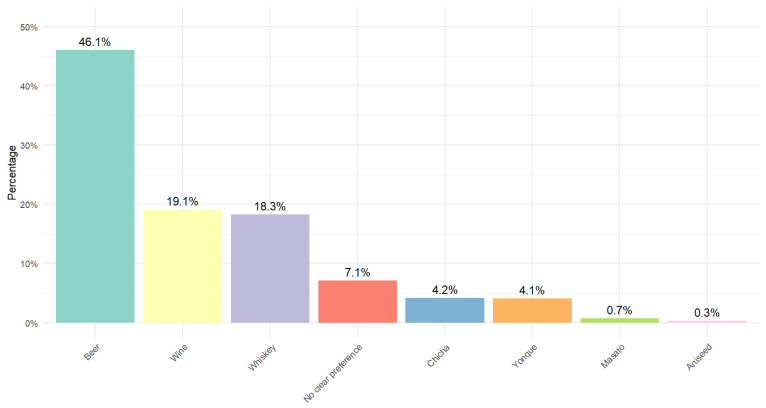
Distribution of alcohol types consumed in the population.

Figure 1 illustrates the distribution of alcoholic beverage preferences in the studied population. Beer is the most popular beverage, consumed by 46.1% of drinkers. Wine and whiskey are the most common choices, at 19.1% and 18.3%, respectively. Traditional drinks such as chicha (4.2%) and yonque (4.1%) have a notable, albeit smaller, presence. Masato (0.7%) and aniseed liquor (0.3%) are the least consumed. 7.1% of participants showed no clear preference for a specific beverage.

**Figure 2:**
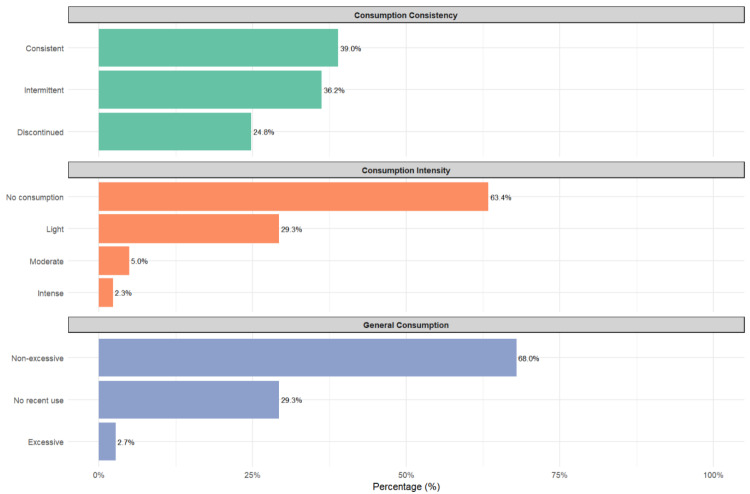
Patterns of alcohol consumption: general use, intensity, and consistency.

Figure 2 presents a comprehensive overview of alcohol consumption patterns in the studied population. Regarding consistency, a relatively balanced distribution is observed, with 39% consistent consumers, 36.2% intermittent consumers, and 24.8% who have discontinued their consumption. The intensity of consumption shows that the majority (63.4%) do not consume alcohol, followed by 29.3% with light consumption, while moderate (5%) and heavy (2.3%) consumption are less prevalent. Regarding overall consumption, 68% exhibit a non-excessive pattern, 29.3% have not consumed recently, and only 2.7% report excessive consumption.

Table 2 presents the regression analysis of alcohol consumption patterns versus types of HTN. Regarding overall consumption, excessive consumption is significantly associated with a higher risk of HTN (aPR: 1.19, 95% CI: 1.07–1.31), IDH (aPR: 1.61, 95% CI: 1.20–2.16), and SDH (aPR: 1.45, 95% CI: 1.18–1.78), compared to no recent consumption. The consumption intensity shows a similar trend, with moderate and heavy consumption associated with a higher risk of various types of HTN, being particularly notable for SDH in moderate consumption (aPR: 1.67, 95% CI: 1.44–1.93).

Regarding consumption consistency, consistent consumption is associated with a higher risk of HTN (aPR: 1.07, 95% CI: 1.02–1.13), IDH (aPR: 1.25, 95% CI: 1.06–1.47), and SDH (aPR: 1.19, 95% CI: 1.08–1.33), compared to discontinued consumption. Concerning types of alcoholic beverages, varied associations are found. Notably, wine consumption shows a protective association against HTN (aPR: 0.81, 95% CI: 0.73–0.91) and ISH (aPR: 0.75, 95% CI: 0.64–0.88). In contrast, beverages such as whiskey, chicha, and masato are associated with a higher risk of various types of HTN, with masato showing the strongest association with HTN (aPR: 1.55, 95% CI: 1.19–2.02) and ISH (aPR: 1.61, 95% CI: 1.15–2.25).

## 4. Discussion

### 4.1. Main Findings

Our study reveals that the association between alcohol consumption and HTN varies according to consumption pattern, intensity, consistency, and beverage type, with differential effects across hypertension subtypes. The main findings indicate that excessive alcohol consumption is significantly associated with a higher risk of HTN, IDH, and SDH. The intensity and consistency of consumption also showed significant associations, with consistent consumption linked to a higher risk of various types of HTN. Notably, we observed differential effects according to the kind of alcoholic beverage consumed: while wine consumption showed a protective association against certain types of HTN, traditional drinks such as chicha and masato were associated with a higher risk. These results underscore the importance of considering not only the quantity but also the pattern and type of alcohol consumption in assessing the risk of HTN, offering an essential approach to prevention and management strategies of this disease in the Peruvian context.

### 4.2. Overall Alcohol Consumption and Its Relationship with Different Types of HTN

Our finding that excessive alcohol consumption is associated with a higher risk of HTN, IDH, and SDH is consistent with previous literature. For instance, a meta-analysis conducted by Roerecke et al. found a dose–response relationship between alcohol consumption and the risk of HTN, with a significant increase in risk even at moderate levels of consumption [16].

The stronger association we observed between excessive consumption and IDH is particularly interesting. This finding aligns with the results of Husain et al., who reported that elevated alcohol consumption has a more pronounced effect on DBP than on SBP [17]. This could be explained by the impact of alcohol on peripheral vascular resistance, a mechanism proposed by Puddey and Beilin [18].

However, it is essential to note that our study did not find a significant association between non-excessive consumption and a higher risk of HTN, which differs from some previous studies. For example, the INTERSALT study, a multinational study, found a linear relationship between alcohol consumption and blood pressure, even at low levels of consumption [19]. This discrepancy could be due to differences in the definition of non-excessive consumption or specific factors in the Peruvian population that moderate this relationship.

### 4.3. Intensity of Consumption and Its Impact

The intensity of alcohol consumption showed a clear association with the risk of HTN in our study, with distinctive patterns for different types of HTN. Our findings indicate that heavy consumption is significantly associated with a higher risk of SDH, consistent with existing literature. For example, a prospective study conducted by Briasoulis et al. found that heavy alcohol consumption (more than four drinks per day) was associated with a significant increase in the risk of HTN in both men and women [20].

Interestingly, we observed a gradual association between the intensity of consumption and the risk of isolated diastolic HTN, with a progressive increase in risk from light to heavy consumption. This finding aligns with the results of Fuchs et al., who reported a dose–response relationship between alcohol consumption and the incidence of HTN in a prospective cohort study [21].

However, it is essential to note that we did not find a significant association between the intensity of consumption and ISH. This contrasts with some previous studies, such as that of Sesso et al., which found a positive relationship between alcohol consumption and SBP [22]. This discrepancy could be due to differences in the characteristics of the studied population or the specific consumption patterns of the Peruvian population.

Our results underscore the importance of considering not only the presence of alcohol consumption but also its intensity in assessing the risk of HTN. Furthermore, they suggest that the effects of alcohol on blood pressure may vary depending on the component (systolic or diastolic) considered, which has important implications for understanding the underlying mechanisms and strategies for the prevention and management of HTN.

### 4.4. Consistency of Consumption and Its Implications

The consistency of alcohol consumption emerged as an essential factor in our study, revealing significant associations with different types of HTN. Our results show that consistent alcohol consumption is associated with a higher risk of HTN, IDH, and SDH compared to discontinued consumption. These findings align with existing literature on the long-term effects of regular alcohol consumption on blood pressure.

A prospective study conducted by Wellman et al. found that regular alcohol consumption over time was associated with a higher risk of developing HTN, even at levels of consumption considered moderate [23]. Our results support this observation, suggesting that continuous exposure to alcohol, even in moderate amounts, may have cumulative effects on blood pressure.

The stronger association we observed between consistent consumption and IDH is particularly interesting. This finding aligns with the results of Ohira et al., who reported that regular alcohol consumption had a more pronounced effect on diastolic blood pressure than on systolic blood pressure in a Japanese cohort [23]. This could be explained by the chronic effects of alcohol on peripheral vascular resistance and endothelial function, as suggested by Piano and Phillips in their review of the cardiovascular effects of alcohol [24].

It is important to note that we did not find a significant association between intermittent consumption and a higher risk of HTN, which differs from some previous studies. For example, Mostofsky et al. found that episodic heavy consumption (binge drinking) was associated with a higher risk of cardiovascular events, including HTN [25]. This discrepancy could be due to differences in the definition of intermittent consumption or specific factors in the Peruvian population that moderate this relationship.

Our findings underscore the importance of considering not only the quantity but also the consistency of alcohol consumption in assessing the risk of HTN. This has important implications for prevention strategies and public health recommendations, suggesting that even regular consumption patterns considered “moderate” could increase the risk of HTN in the long term.

### 4.5. Specific Types of Alcoholic Beverages and Their Differential Effects

In our study, we observed significant differential effects according to the type of alcoholic beverage consumed, which adds an essential layer of complexity to the relationship between alcohol consumption and HTN. Notably, we found that wine consumption was associated with a lower risk of undiagnosed HTN and isolated systolic HTN. This finding is consistent with several previous studies, such as that of Chiva-Blanch et al., which suggests that polyphenolic compounds present in red wine, especially resveratrol, could have protective cardiovascular effects [26]. However, it is important to interpret these results cautiously, as other factors, such as lifestyle associated with wine consumption, could influence this relationship.

In contrast, we observed that consumption of distilled spirits like whiskey and traditional Peruvian beverages such as chicha and masato were associated with a higher risk of various types of HTN. This aligns with the findings of Sesso et al., who reported that liquor consumption was more strongly related to the risk of HTN compared to wine or beer [22]. The robust association between masato consumption and the risk of HTN and IDH is a novel finding that warrants further investigation.

Interestingly, beer consumption showed a weaker association with the risk of HTN compared to other beverages, which differs from some previous studies. For example, Zilkens et al. found that beer consumption had a more pronounced effect on blood pressure than wine [27]. This discrepancy could be due to differences in consumption patterns or the composition of beers consumed by the Peruvian population.

Several factors could explain the association between beer and SDH. According to a study by Sierksma et al., beer contains significant amounts of polyphenols and other bioactive compounds that could influence blood pressure [28]. Additionally, it has been suggested that the high carbohydrate content in beer could contribute to its effect on blood pressure [29,30]. These components, along with the alcohol content, could act synergistically to affect both types of pressures.

Regarding chicha and yonque, traditional Peruvian beverages, their effect similar to beer on SDH is particularly intriguing. Although there is less research on these beverages, we can speculate about possible mechanisms. The variable, but potentially high, alcohol content of both drinks could be a main factor. Additionally, it is notable that chicha de jora seems to have a broader effect, increasing the risk of all types of HTN. This could be due to a combination of factors. The variability in the composition of chicha, which can differ significantly in its alcohol content and other compounds depending on the preparation method, could explain its more generalized effect. Moreover, chicha consumption patterns might vary from other beverages, possibly with more frequent intake or in larger quantities. Socioeconomic and cultural factors associated with chicha consumption could also play an important role, perhaps correlated with other risk factors for HTN that we have not been able to fully control for in our study, which could also generate compounds that affect blood pressure similarly to beer. However, specific studies on its cardiovascular health effects are still needed [31].

These findings underscore the need for further investigation into the specific components of these traditional beverages and their effects on cardiovascular health. They also suggest that public health recommendations should consider not only alcohol content but also other beverage components and cultural consumption patterns. The complexity of these results highlights the importance of a holistic approach in research on alcohol consumption and cardiovascular health, especially in contexts with a rich diversity of traditional beverages.

These results emphasize the importance of considering not only the quantity but also the type of alcoholic beverage in assessing the risk of HTN. They suggest that public health recommendations on alcohol consumption should take these differences into account, especially in contexts where traditional drinks play an important role in local culture.

### 4.6. Importance of the Study for Public Health

Our findings have relevant implications for public health in Peru. The results indicate that prevention strategies should consider not only the quantity of alcohol consumed but also the frequency, intensity, and type of beverage. The significant associations found for traditional beverages such as chicha and masato are particularly important, as these drinks are culturally embedded in Peruvian society yet have received limited attention in cardiovascular risk assessments. Public health messages should incorporate specific information about these beverages while respecting their cultural significance.

The apparent protective effect of wine observed in our study should be interpreted with caution and communicated carefully to avoid inadvertently promoting increased alcohol consumption. Additionally, given that different beverage types and consumption patterns are associated with distinct hypertension subtypes, clinical assessments could benefit from more detailed evaluation of patients’ alcohol consumption habits, potentially leading to more personalized prevention strategies.

### 4.7. Limitations and Strengths

This study has several limitations that should be considered when interpreting the results. The cross-sectional design limits our ability to establish causal relationships between alcohol consumption and HTN. The reliance on self-reported data on alcohol consumption may introduce recall and social desirability biases. Additionally, we lacked detailed information on the duration of alcohol consumption over the lifetime, which could provide a more complete picture of cumulative alcohol exposure. Furthermore, we did not conduct stratified analyses by sex, age groups, or geographic region to explore potential effect modification, as this would substantially increase the number of comparisons given the multiple exposures and outcomes evaluated. Future studies specifically designed to test effect modification hypotheses could address these questions.

## 5. Conclusions and Recommendations

The findings underscore the importance of considering the quantity, frequency, intensity, and type of alcoholic beverage consumed when assessing cardiovascular risk. The differential associations observed between traditional drinks and various types of HTN highlight the need for culturally sensitive approaches to prevention and control strategies.

Based on these findings, future public health guidelines in Peru should incorporate specific messages about the risks associated with different consumption patterns and beverage types, including traditional drinks. Healthcare professionals should consider assessing not only the quantity but also the type and frequency of alcohol consumption. Longitudinal studies are needed to establish causal relationships and explore the biological mechanisms underlying the differential effects of various alcoholic beverages on blood pressure.

## Figures and Tables

**Table 1:** Characteristics of the study sample.

Characteristic	n = 236,243
Sex	
Female	104,054 (44.05%)
Male	132,188 (55.95%)
Age	41.06 (16.34)
Residence	
Urban	186,424 (78.91%)
Rural	49,819 (21.09%)
Education	
No Level	480 (0.22%)
Primary	46,169 (21.22%)
Secondary	95,459 (43.88%)
Superior	75,420 (34.67%)
Wealth Index	
The poorest	48,680 (20.61%)
Poor	48,891 (20.70%)
Medium	47,871 (20.26%)
Rich	46,482 (19.68%)
Richest	44,320 (18.76%)
Altitude	1048.99 (1360.44)
Systolic arterial pressure	121.09 (16.31)
Diastolic arterial pressure	73.19 (9.86)
Smoking status	
Has never smoked	185,299 (78.44%)
Former smoker	21,717 (9.19%)
Currently smoker	25,009 (10.59%)
Daily smoker	4218 (1.79%)
HTN	
No	180,698 (86.85%)
Yes	27,362 (13.15%)
ISH	
No	218,590 (92.53%)
Yes	17,653 (7.47%)
IDH	
No	232,158 (98.27%)
Yes	4085 (1.73%)
SDH	
No	227,786 (96.42%)
Yes	8457 (3.58%)
n (%); Mean (SD)	

**Table 2:** Regression analysis of alcohol consumption patterns and types of HTN.

Characteristic	HTN		ISH		IDH		SDH	
	aPR *	95% CI	aPR *	95% CI	aPR *	95% CI	aPR *	95% CI
General consumption								
No recent use	Ref.		Ref.		Ref.		Ref.	
Non-excessive	0.99	0.95–1.04	0.97	0.91–1.03	1.1	0.96–1.27	1.06	0.96–1.16
Excesive	1.19	1.07–1.31	0.98	0.83–1.15	1.61	1.20–2.16	1.45	1.18–1.78
Consumption Intensity								
No consumption	Ref.		Ref.		Ref.		Ref.	
Light	1.03	0.99–1.08	0.99	0.93–1.06	1.15	1.03–1.30	1.06	0.97–1.16
Moderate	1.28	1.18–1.38	1.07	0.95–1.20	1.25	1.01–1.59	1.67	1.44–1.93
Intense	1.26	1.13–1.41	1.03	0.87–1.22	1.71	1.29–2.27	1.49	1.23–1.82
Consumption Consistency								
Discontinued	Ref.		Ref.		Ref.		Ref.	
Intermittent	0.97	0.92–1.02	0.96	0.90–1.03	1.04	0.89–1.23	1.01	0.91–1.13
Consistent	1.07	1.02–1.13	0.98	0.92–1.06	1.25	1.06–1.47	1.19	1.08–1.33
Alcohol types consumed								
No consumption	Ref.		Ref.		Ref.		Ref.	
Beer	1.04	0.97–1.12	0.98	0.88–1.09	1.02	0.84–1.23	1.17	1.02–1.34
Wine	0.81	0.73–0.91	0.75	0.64–0.88	1.13	0.84–1.49	0.8	0.63–1.02
Whiskey	1.18	1.06–1.32	1.03	0.88–1.22	1.56	1.18–2.05	1.23	0.99–1.54
Chicha	1.28	1.12–1.47	1.27	1.05–1.54	1.7	1.15–2.49	1.16	1.02–1.54
Yonque	1.15	1.00–1.30	1.11	0.92–1.34	0.92	0.64–1.34	1.4	1.09–1.82
Masato	1.55	1.19–2.02	1.61	1.15–2.25	1.53	0.64–3.67	1.51	0.88–2.58
Anissed	1.07	0.64–1.80	0.76	0.40–1.45	2.25	0.81–6.24	1.23	0.35–4.27
No clear preference	1.15	0.99–1.34	1.09	0.88–1.37	1.54	1.07–2.21	1.08	0.79–1.47

* Each factor has been independently adjusted for sex, age (RCS), natural region, area of residence, wealth index, smoking status, and altitude (RCS). HTN: Hypertension; ISH: Isolated Systolic Hypertension; IDH: Isolated Diastolic Hypertension; SDH: Systolic-Diastolic Hypertension; aPR: Adjusted Prevalence Ratio; 95% CI: 95% Confidence Interval; RCS: Restricted Cubic Splines; Ref.: Reference category.

## Data Availability

The data supporting the findings of this study can be accessed by the original research paper at the follow link: https://proyectos.inei.gob.pe/microdatos/ (accessed on 10 October 2025).
